# Unlocking *KRAS*: Navigating Its Molecular Biology and Treatment Landscape Among Gastrointestinal Malignancies

**DOI:** 10.3390/curroncol33030148

**Published:** 2026-03-03

**Authors:** Austin Frisch, Eric Martin, Timothy Cannon, Raymond Wadlow, Srivatsan Raghavan, Triparna Sen, Nagla Abdel Karim

**Affiliations:** 1 Inova Fairfax Department of Internal Medicine, Inova Fairfax Hospital, Fairfax, VA 22042, USA; 2 Inova Schar Cancer Center, Inova Fairfax Hospital, Fairfax, VA 22042, USAraymond.wadlow@inova.org (R.W.); 3 Department of Medical Oncology, Dana-Farber Cancer Institute, Boston, MA 02215, USA; 4 Harvard Medical School, Boston, MA 02115, USA; 5 Department of Internal Medicine, The Ohio State University, Columbus, OH 43210, USA; triparna.sen@osumc.edu; 6 George Washington (GW) Cancer Center, Washington, DC 20037, USA

**Keywords:** *KRAS*, gastrointestinal malignancy, pancreatic cancer, colorectal cancer, resistance

## Abstract

*KRAS*-targeted therapy has opened new doors in the world of oncology, and many trials are underway for *KRAS* specific treatments for gastrointestinal (GI) malignancies. Outlining the current state of *KRAS* therapy and the remaining research gaps pertaining to these deadly cancers is crucial for the development of future therapeutics. In this review, we focus on the relationship between *KRAS* and GI malignancies. Current therapies are discussed with an in-depth exploration of the *KRAS* gene and how it connects to pancreatic, colorectal and other GI malignancies. Promising clinical trials and future therapies are highlighted while discussing the molecular biology behind them. Specifically, trials focusing on upcoming *KRAS* on and off inhibitors in development as well as variant focused inhibitors targeting the more common mutations G12D and G12V. We discuss exciting new pan/multi *KRAS* inhibitors that have been successful in pre-clinical trials. More unique therapeutic options include *KRAS* T cell therapies, vaccines, and combination strategies with immunotherapy. Furthermore, we address the difficulties with *KRAS* therapy, and the potential future directions needed to overcome them. An in-depth current literature review was done along with a review of the active clinical trials for *KRAS*-targeted therapeutics involving GI malignancies.

## Simple Summary

Kirsten Rat Sarcoma Viral Oncogene (*KRAS*) is one of the most commonly mutated cancer genes in gastrointestinal cancers, especially pancreatic and colorectal cancer. For many years, *KRAS* was considered “undruggable,” meaning it could not be directly targeted with medications. Recently, new drugs have been developed that can block specific *KRAS* mutations, leading to promising results in some patients. However, responses are often temporary because tumors adapt and develop resistance. The effectiveness of *KRAS*-targeted therapies depends on the specific mutation, the type of cancer, and the tumor’s surrounding environment. This review explains how *KRAS* works, summarizes current and emerging treatments, and discusses why some therapies succeed while others fail. We highlight the importance of combining *KRAS* inhibitors with other treatments and tailoring therapy to the specific biology of each cancer. Continued research is needed to improve long-term outcomes for patients with *KRAS*-mutated gastrointestinal cancers.

## 1. Introduction

Kirsten Rat Sarcoma Viral Oncogene (*KRAS*) is an oncogenic driver gene that comes from a family of genes whose protein products drive cell growth and survival. Mutations in the *RAS* gene family are common across malignancies, with *KRAS* being the most mutated isoform of *RAS*. Specifically, within gastrointestinal (GI) malignancies, *KRAS* is mutated in close to 90% of pancreatic ductal adenocarcinoma (PDAC) cases and around 40–50% of colorectal cancer (CRC) cases [1]. Although these two diseases attract the most attention when discussing *KRAS*, many other GI malignancies are known to harbor *KRAS* alterations but at a lower frequency. Researchers have spent years trying to unlock therapeutic strategies against *KRAS*, a target that until recently was coined “undruggable”. Recent successes in targeting malignancies with *KRAS* G12C alterations are encouraging, although this *KRAS* mutation subtype is found in a relatively low proportion of *KRAS*-mutated GI malignancies. Fortunately, *KRAS* G12C targeted agents are just the beginning, and we are entering a new era of precision medicine research that will hopefully lead to more breakthrough therapies for *KRAS*-mutated GI cancers.

Despite the rapidly expanding literature on *KRAS* biology and targeted therapeutics, most existing reviews focus either on *KRAS* as a historically undruggable oncogene or on the clinical development of *KRAS* G12C inhibitors, largely extrapolated from non-small cell lung cancer. What remains insufficiently addressed is how *KRAS* mutation subtype, co-mutation landscape, and tumor microenvironment collectively determine therapeutic vulnerability and resistance in gastrointestinal malignancies. This review aims to fill that gap by integrating differences across *KRAS* alleles with GI-specific biology, resistance mechanisms, and emerging therapeutic classes. Along with discussing individual agents, we emphasize why certain *KRAS*-targeted strategies succeed or fail in PDAC versus colorectal cancer and outline a framework for prioritizing allele-specific, pan-*KRAS*, and combination approaches.

## 2. Molecular Biology of *KRAS*

Cellular division and differentiation are complex processes that rely on specific signaling proteins. A special group of these proteins are encoded by three major genes: *KRAS*, *HRAS*, and *NRAS*. RAS proteins trigger these intracellular signaling pathways by alternating between GTP-bound active and GDP-bound inactive states [2]. They are part of a protein signaling family called membrane bound GTPases and their actions are commonly referred to as being a “molecular switch”. Activation of RAS causes a conformational change in its structure allowing it to drive a cascade of downstream protein interactions, eventually leading to cell proliferation and/or differentiation [3]. The Mitogen-Activated Protein Kinase (MAPK)/extracellular signal-regulated kinase (ERK) signaling pathway responsible for cell cycle regulation and cell proliferation is one of the main pathways influenced by RAS and, therefore, a key inducer of tumorigenesis when RAS proteins are mutated [4].

The *KRAS* gene, located on chromosome 12, is responsible for expressing the KRAS GTPase molecular switch protein, an important mediator of the MAPK/ERK pathways which control cellular division and proliferation. The G domain and the C terminal hypervariable region are two main regions of the *KRAS* gene, with the G domain containing roughly 165 codons including the switch regions responsible for protein conformational changes upon GTP binding [5]. Mutations in the early codon region and different splice variants of this gene have sparked interest as targets for possible therapy [6,7]. Mutations typically occur upstream at codons 12, 13, and 61, with codon 12 having the most frequent amino acid missense mutations including G12D, G12C, G12V, G12R, and G12A among others. *KRAS* mutations in part disrupt GTPase activity and prevent this molecular switch from turning off, leading to constitutive activation of downstream signaling pathways.

**Figure 1:**
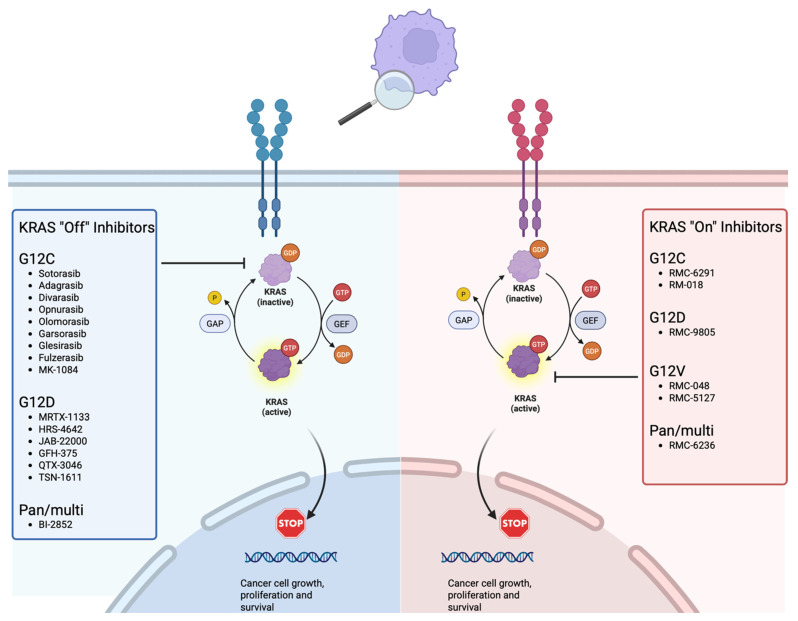
This figure depicts the *KRAS* On/Off Inhibitor mechanism. The *KRAS* pathway is triggered by growth factors binding to receptor tyrosine kinases (represented as the blue and red cell surface receptors) which facilitates dimerization, phosphorylation and GDP release with GTP loading leading to KRAS-GDP inactive (light purple) vs. KRAS-GTP active (dark purple) states. *KRAS* off and on inhibitors block different steps of this cycle by either locking KRAS in its inactive state preventing GTP loading (off) or preventing GTP unloading as well as preventing interaction with downstream proteins (on). Both of these mechanisms prevent signal translocation to the nucleus (arrow leading to STOP sign) thus halting the cancer cell growth, proliferation and survival. Created in BioRender. Frisch, A. (2025) https://BioRender.com/el3m0co (URL accessed on 30 July 2025).

Targeting *KRAS* therapeutically was once thought to be undruggable due to its structure and the intense affinity between GTP and *KRAS* [8], but recent advances in our understanding of *KRAS* structure-function and in small molecule design have led to breakthroughs in the development of different types of *KRAS* therapies [7,9]. *KRAS* inhibitors whose mechanism locks the protein in its GDP-bound state are referred to as “off” inhibitors and constitute most inhibitors in development. In contrast, *KRAS* “on” inhibitors trap the *KRAS* protein in its continuously active GTP-bound state preventing the GTPase molecular switch from oscillating between the RAS-ON/RAS-OFF states and halting signaling [10]. There is growing evidence that these newer “on” inhibitors could be used in combination with “off” inhibitors or other therapies to limit the drug resistance seen in first generation *KRAS* “off” inhibitor monotherapy [11]. Figure 1 highlights the mechanistic distinction between *KRAS* “off” (GDP-state selective) and “on” (GTP-state selective) inhibition, providing a conceptual framework for understanding how nucleotide-state dependency shapes therapeutic activity and resistance. By illustrating how first-generation G12C inhibitors rely on *KRAS* cycling into the inactive GDP-bound state, the figure contextualizes mechanisms of adaptive MAPK reactivation while also explaining the rationale for *RAS*(ON) inhibitors designed to target persistently active GTP-bound *KRAS*. While the only two FDA approved *KRAS* therapies are “off” inhibitors, numerous clinical trials are underway with different mechanistic approaches to targeting *KRAS*.

### 2.1. Structure

Different *KRAS* G12 variants produce distinct structural and biochemical consequences that drive divergent signaling behaviors, therapeutic vulnerabilities, and clinical outcomes. For example, G12C replaces glycine with cysteine, whose thiol side chain occupies a switch-II pocket and forms irreversible covalent bonds with inhibitors while retaining intrinsic GTPase activity and thus maintaining the GDP (off) state and creating a pharmacologic target with “G12C-off” inhibitors [12,13]. In contrast, G12D introduces a negatively charged aspartate in the nucleotide-binding/switch region, altering local nucleotide interactions and increasing steady-state GTP loading [14]. G12V substitutes a bulkier hydrophobic valine that alters the P-loop/switch interfaces, resulting in lower intrinsic GTPase activity and more persistent GTP-bound (constitutively active) signaling. G12C tumors are uniquely druggable with approved covalent inhibitors such as sotorasib and adagrasib, particularly in non-small-cell lung cancer, whereas G12D and G12V lack approved allele-selective drugs as of 2025 despite being the dominant *KRAS* variants in GI malignancies [15].

### 2.2. Co-Mutations

Meanwhile, co-occurring alterations—including *TP53*, *SMAD4*, *STK11*, and *KEAP1*—further shape the biology and therapeutic response of *KRAS*-mutated gastrointestinal malignancies; for example, in PDAC, concurrent *TP53*, *CDKN2A*, *ARID1A* and *SMAD4* mutations alongside *KRAS* are linked to higher tumor mutational burden, immunologically colder micro-environments, increased metastatic potential and worse overall survival [15,16]. In colorectal cancer, specific co-mutations alongside *KRAS* significantly influence treatment response and outcomes: for example, CRC patients with concurrent *KRAS* and TP53 mutations demonstrated markedly lower objective responses to standard first-line chemotherapy and higher rates of recurrence and metastasis compared to those without the double-mutation [17]. A recent study of Chinese CRC patients found that *KRAS* mutations combined with APC mutations were associated with increased tumor mutational burden (TMB)—notably highest with *KRAS* G12V plus APC—suggesting that the co-mutation context may inform immune-checkpoint inhibitor responsiveness [18].

### 2.3. KRAS Amplification and Copy Number Alterations

*KRAS* copy number alterations (CNAs), including focal amplification, have been described in colorectal and pancreatic cancers and represent a potential modifier of *KRAS*-driven tumor biology [19,20,21,22]. Amplification increases total *KRAS* transcript and protein abundance—often including the mutant allele—which may augment downstream MAPK signaling output and increase signaling flux in preclinical and translational models [19,20]. Emerging data further suggest that *KRAS* amplification can function as a mechanism of acquired resistance to allele-selective *KRAS* inhibition, including *KRAS* G12C inhibitors, by increasing target abundance and restoring pathway activity despite continued drug exposure [22,23]. In some resistant tumors, amplification appears alongside other adaptive signaling changes, including receptor tyrosine kinase activation and feedback pathway engagement, although the frequency and clinical impact of these co-occurring events remain incompletely defined [23]. Collectively, these observations provide a biologically plausible rationale for incorporating *KRAS* copy number status into biomarker analyses and for exploring broader pathway suppression strategies—such as pan-*KRAS* or pan-RAS inhibition—in amplification-driven disease contexts [24].

### 2.4. PDAC Subtypes and KRAS

In PDAC, there is well-documented phenotypic heterogeneity that broadly falls into two defined epithelial states: the “classical” subtype and the “basal-like” (or quasi-mesenchymal) subtype. The classical subtype is characterized by higher expression of differentiation markers such as GATA6, glandular/epithelial morphology, and lower *KRAS* pathway dependence (lower p-ERK and fewer *KRAS*-target gene signatures) compared with basal-like cells [19,20]. In contrast, the basal-like subtype displays more invasive and less differentiated morphology, higher *KRAS* effector activity, poorer prognosis, and greater chemoresistance [19]. Regarding response to *KRAS*-targeted therapy, preclinical work shows that basal-like PDAC cells are more sensitive to direct *KRAS* inhibition (for example, in models of the *KRAS*-G12D inhibitor MRTX1133) than the classical subtype [21]. 

## 3. Current and Evolving *KRAS*-Targeted Therapies in GI Malignancies

Therapeutic targeting of *KRAS* in gastrointestinal malignancies has rapidly evolved from a longstanding scientific challenge to a clinically actionable strategy, driven by advances in structural biology and drug design. This section reviews allele-specific inhibitors, pan-*KRAS* and RAS(ON) platforms, immunotherapeutic approaches, and rational combination strategies, emphasizing differences in clinical maturity and disease-specific applicability across PDAC and colorectal cancer. To contextualize the clinical maturity of evolving *KRAS*-targeted strategies across gastrointestinal malignancies, detailed trial landscapes are summarized in Supplementary Tables S1–S4.

### 3.1. KRAS G12C Inhibitors

Sotorasib and adagrasib—two *KRAS* G12C “off” inhibitors—have demonstrated meaningful clinical activity in NSCLC, where *KRAS* G12C occurs in approximately 13% of cases [25,26]. In gastrointestinal malignancies, G12C mutations are less common—occurring in approximately 1–3% of PDAC and approximately 4% of metastatic colorectal cancer (mCRC) [27,28]. In the CodeBreaK 100 trial, 38 patients with previously treated PDAC receiving sotorasib achieved an objective response rate (ORR) of 21%, with a median progression-free survival (mPFS) of 4.0 months and median overall survival (OS) of 6.9 months; grade ≥3 treatment-related adverse events (TRAEs) occurred in six patients [27]. In the KRYSTAL-1 study, adagrasib demonstrated an ORR of 33%, mPFS of 5.4 months, and OS of 8.0 months in 21 pretreated PDAC patients [26].

In mCRC, sotorasib monotherapy achieved an ORR of 9.7% and mPFS of 4.0 months among 62 patients, while adagrasib demonstrated an ORR of 19% and mPFS of 5.6 months in 44 patients, with grade ≥3 TRAEs reported in 15 patients [25,29,30]. Adaptive EGFR-mediated MAPK reactivation is a well-established resistance mechanism to *KRAS* inhibition in CRC, providing a rationale for vertical pathway blockade [31]. Preclinical and translational studies further support dual *KRAS* and EGFR inhibition to enhance pathway suppression and delay resistance [32,33,34].

In KRYSTAL-1, adagrasib combined with cetuximab improved outcomes in mCRC, achieving an ORR of 34% and mPFS of 6.9 months, compared with historical monotherapy results of approximately 19% ORR and 5–6 months mPFS [35]. Similarly, in CodeBreaK 101, sotorasib combined with panitumumab demonstrated an ORR of approximately 30% in 40 patients with *KRAS* G12C–mutant CRC, with grade ≥3 TRAEs occurring in approximately 27% of patients [36]. These findings are consistent with broader clinical observations of EGFR-driven adaptive resistance in CRC and support continued evaluation of combination strategies [37,38,39]. While adagrasib has demonstrated numerically higher response rates than sotorasib in some CRC cohorts—potentially related to its longer half-life and sustained target inhibition—monotherapy activity remains limited in this disease [40]. Combination strategies with EGFR antibodies approximately double response rates and extend PFS by approximately 2–3 months, supporting the results seen in BRAF V600E–mutant CRC in the BEACON study and supporting ongoing phase III development [30,32]. Although toxicity increases with combination therapy, adverse events have remained manageable across studies [38]. An overview of ongoing and late-phase *KRAS*-targeted clinical trials in non-PDAC gastrointestinal malignancies, primarily colorectal cancer, is summarized in Supplementary Table S1.

Next-generation *KRAS* G12C inhibitors aim to improve potency, selectivity, and target engagement in GI tumors. Glecirasib has demonstrated encouraging activity across *KRAS* G12C–mutant solid tumors, including PDAC and CRC cohorts. In early-phase studies, response rates have varied by tumor type and treatment context, with ORRs in the low- to mid-20% range reported for monotherapy in heavily pretreated CRC cohorts and higher response rates observed in smaller PDAC cohorts [41,42]. Divarasib achieved an ORR of 29% and mPFS of 5.6 months in 55 patients with mCRC, increasing to 36% ORR and 6.9 months mPFS in the 400 mg daily cohort [43,44]. Additional emerging agents—including MK-1084, olomorasib, and garsorasib—are being evaluated alone and in combinations such as with cetuximab or SHP2 inhibition. In combination regimens, response rates have approached or exceeded 40% in early-phase studies, with median PFS extending toward 7–8 months in select cohorts [45,46,47,48,49,50]. Collectively, these agents demonstrate improved pharmacologic profiles and more sustained pathway suppression, suggesting the potential to overcome limitations observed with first-generation inhibitors. Nonetheless, combination strategies will likely remain necessary to maximize clinical benefit in *KRAS* G12C–mutant GI malignancies.

Take-Home Message:

Clinical Maturity:

Most advanced among *KRAS*-directed strategies. Phase II–III data available in CRC and PDAC; regulatory approval established in NSCLC and under active evaluation in GI malignancies.

Likelihood of Regulatory Approval in GI Cancers:

High in mCRC when combined with EGFR blockade; moderate in PDAC given modest monotherapy activity.

Expected Role in Treatment:

Likely second-line or later therapy in *KRAS* G12C–mutant mCRC as part of dual *KRAS*–EGFR blockade. In PDAC, potential role in later-line settings or combination backbone pending durability data. Monotherapy unlikely to be sufficient in CRC.

### 3.2. KRAS G12D Inhibitors in Development

*KRAS* G12D mutations are highly represented in both pancreatic cancer (40%) and CRC (30–35%) [51]. Due to a putative “colder” nucleophile region as compared to the G12C variant [52], inhibitors against *KRAS* G12D were initially more challenging to develop [51]. The G12D inhibitor MRTX1133 uses complex binding properties and nanomolar affinity to bind to GDP-bound *KRAS* G12D causing a halt in downstream signal transduction [53,54]. There is also growing evidence that MRTX1133 can help remodel the tumor microenvironment (TME) by modulating cancer-associated fibroblast (CAF) and lymphocyte populations [55,56]. This drug is currently in a clinical study evaluating its safety and efficacy in *KRAS* G12D mutated solid tumors (NCT05737706) [55,56]. Other *KRAS* G12D inhibitors currently recruiting in clinical trials include RMC-9805 (NCT06040541), HRS-4642 (NCT05533463), GFH375 (NCT06500676), QTX3046 (NCT06428500), and TSN1611 (NCT06385925), among others. Results from a phase 1 trial of patients taking RMC-9805 showed an objective response rate (ORR) of 30% and a disease control rate (DCR) of 80% [57]. GFH375 achieved an ORR of approximately 27% among a group that included PDAC and CRC patients [58].

Selective protein degradation is another technique being explored to target mutated RAS proteins. Proteolysis targeting chimeras (PROTAC) consist of a two-segment molecule used to facilitate proteosome-dependent degradation of its target [59]. The tumor targeting *KRAS* degrader (TKD) is one such molecule that has been developed for the targeting and degradation of *KRAS* mutants and has been shown to eliminate different *KRAS* variants in preclinical studies [60]. ACBI3 is another PROTAC degrader that showed promise in vivo with degradation of 13/17 main *KRAS* mutations with the goal of suppressing *KRAS* mutants altogether [61]. These novel approaches raise further optimism for more effective strategies to control RAS-mutant cancers. ASP3082 represents the first clinical *KRAS* G12D protein degrader evaluated in patients with advanced solid tumors, including PDAC, with early-phase results demonstrating acceptable safety and favorable pharmacokinetics [62]. ARV-806, a next-generation PROTAC designed to degrade mutant *KRAS* G12D, is currently undergoing phase I/II evaluation, with trial data focused on dose optimization and biomarker readouts rather than confirmed efficacy endpoints [63].

Despite enthusiasm surrounding *KRAS* G12D inhibitors such as MRTX1133, several translational barriers temper expectations for rapid clinical impact in PDAC. The dense desmoplastic stroma, elevated interstitial pressure, and poor vascularization characteristic of pancreatic tumors may substantially limit uniform intratumoral drug delivery, even when systemic target engagement is achieved. As a result, incomplete spatial *KRAS* suppression could permit residual signaling sufficient to sustain tumor survival. In addition, sustained *KRAS* inhibition in PDAC appears to promote adaptive transcriptional reprogramming and lineage plasticity—mechanisms increasingly recognized as central resistance drivers in *KRAS*-dependent tumors—thereby limiting durability of response [21]. Although preclinical models demonstrate deep and selective *KRAS* G12D suppression with marked tumor regression, these responses are frequently transient. Resistance commonly emerges through MAPK pathway reactivation, compensatory PI3K or parallel survival signaling, and non-genetic transcriptional adaptation that restores oncogenic output despite continued drug exposure [21,24,64]. These findings suggest that biochemical potency alone may not translate into durable pathway extinction in the complex PDAC ecosystem. Early, first-in-human data with agents such as RMC-9805 confirm the feasibility of directly targeting *KRAS* G12D and demonstrate encouraging disease control; however, response durability, optimal dosing strategies, and dominant resistance trajectories remain incompletely defined [24,57].

Furthermore, *KRAS* G12D plays a central role in shaping the immunosuppressive, metabolically constrained PDAC microenvironment, influencing stromal composition, cytokine signaling, and immune exclusion [37,65,66,67]. While pathway inhibition may partially remodel this environment, it is uncertain whether G12D blockade alone can generate sustained immune activation or overcome stromal barriers. Collectively, these tumor-intrinsic and microenvironmental constraints suggest that *KRAS* G12D inhibitors—while representing a major scientific breakthrough—are unlikely to produce durable benefit as monotherapy. Instead, they will more plausibly function as foundational components of rational combination strategies that simultaneously address adaptive signaling, stromal resistance, and immune suppression in *KRAS*-driven PDAC.

Take-Home Message:

Clinical Maturity:

Early clinical development (Phase I/II). Strong preclinical validation; first-in-human efficacy signals emerging.

Likelihood of Regulatory Approval:

Moderate but dependent on durability and combination feasibility. Regulatory pathway is contingent on demonstrating sustained benefit in PDAC.

Expected Role in Treatment:

Overall, the role these inhibitors may play in PDAC remain unclear. The RMC-9805 inhibitor seems to be very well tolerated making monotherapy a possibility. However, the most plausible future role is likely as a combination backbone—potentially paired with stromal modulation, immune therapy, or downstream pathway inhibition—particularly in earlier metastatic lines or high-risk minimal residual disease settings.

### 3.3. KRAS G12V Therapies

*KRAS* G12V is seen in roughly 30% of PDAC cases and close to 25% of CRC cases making it another desirable target for *KRAS* based therapy. Two G12V inhibitors have shown promise in pre-clinical trials and xenograft models: RMC-5127 and RM-048. RMC-5127 showed anti-tumor activity in PDAC and CRC models in vivo [68]. Similarly, RM-048 proved effective at blocking RAS signaling and is currently being tested in pre-clinical studies in combination with other *KRAS* inhibitors including RMC-6236 [69]. While we wait for these and other G12V inhibitors to progress along in future trials, active trials involving T cell therapy that use G12V as a target are ongoing. A phase 1 trial studying dosing and side effects is underway for a T cell-based therapy that uses CD8 and CD4 cells with receptors that have high affinity to G12V [70]. AFNT-211 is a therapy currently in an active clinical trial that uses engineered T cells that recognize malignant cells harboring *KRAS* G12V alterations, where patients undergo leukapheresis to collect T cells for AFNT-211 manufacturing and then receive lymphodepleting chemotherapy prior to the infusion of AFNT-211 [71].

*KRAS* G12V presents translational challenges that differ meaningfully from other alleles and should not be approached through direct extrapolation from G12C development paradigms. Biochemically, G12V significantly impairs intrinsic and GAP-mediated GTP hydrolysis, resulting in a higher steady-state fraction of GTP-bound active *KRAS* compared with wild-type protein. This shift toward sustained GTP loading may limit the proportion of *KRAS* in the GDP-bound conformations required for inhibitors that depend on inactive-state binding, suggesting that effective therapeutic strategies may require active-state engagement or broader pathway suppression. Such approaches raise important considerations regarding depth of inhibition and therapeutic window in *KRAS*-driven GI tumors.

Clinically, G12V-mutant cancers—particularly in PDAC—arise within a genomic context frequently characterized by concurrent alterations in TP53, CDKN2A, and SMAD4, which may attenuate the impact of single-pathway targeting [21]. Even if direct *KRAS* G12V inhibition proves feasible, durable benefit will likely depend on addressing parallel survival networks and adaptive transcriptional programs that sustain tumor fitness under therapeutic pressure [22,23,72,73,74]. These factors suggest that G12V-directed therapy will require rational combination strategies and biomarker-guided patient selection rather than reliance on monotherapy efficacy.

Finally, because meaningful suppression of G12V-driven signaling may necessitate active-state or pan-*KRAS* approaches, the balance between adequate oncogenic blockade and preservation of wild-type RAS signaling remains uncertain [24]. Defining this therapeutic window—particularly in PDAC—will be critical to determining whether G12V inhibition can translate into a clinically meaningful and tolerable benefit. A summary of allele-specific *KRAS*-directed clinical trials in PDAC is provided in Supplementary Table S2.

Take-Home Message:

Clinical Maturity:

Preclinical to early-phase clinical development. Limited direct inhibitor data; TCR-based therapies in early trials.

Likelihood of Regulatory Approval:

Uncertain and longer-term. Dependent on demonstration of druggable conformations or successful cellular immunotherapy platforms.

Expected Role in Treatment:

Potential niche application in molecularly selected patients. Cellular therapies may be positioned in refractory disease or specialized centers. Small-molecule G12V inhibitors, if successful, would likely follow a combination paradigm similar to G12D.

### 3.4. Pan-RAS/KRAS Inhibitors in Development

Clinical trials are currently underway to test therapies that block multiple RAS proteins simultaneously. Broad inhibition of numerous RAS subtypes using a single therapeutic agent will ideally extend survival while preventing or delaying the development of resistance. Daraxonrasib, also known as RMC-6236, is an exciting pan-RAS inhibitor that showed an ORR of 38% in a phase 1 study [24]. It forms a tri-complex inhibitor by binding the RAS protein, cyclophilin A, and a chaperone protein which then blocks RAS signaling downstream [24]. RMC-6236 is currently being tested in a phase 3 study in patients with second line metastatic PDAC against standard of care chemotherapy. An updated analysis on 68 PDAC patients harboring a *KRAS* mutation in the second line setting being treated with RMC-6236 had a mPFS of 8.5 months and a mOS of 14.5 months [75]. Treatment-related adverse events led to dose modifications in 35% of patients with 22% of patients experiencing grade 3 or higher events, but no patient needed to discontinue treatment. In addition to pan-RAS inhibitors, several pan-*KRAS* inhibitors that simultaneously target different mutated alleles are in early development. BI-2865 has shown anti-tumor activities in *KRAS*-mutated cancer cell lines [76]. LY4066434, another pan-*KRAS* inhibitor, is being investigated for safety and tolerability in phase I trials (NCT06607185). The variety of pan-*RAS*/*KRAS*-mutant inhibitor trials that are currently ongoing and early promising pre-clinical trials raise hope that some of these agents may be approved in the near future for GI cancer patients with RAS-mutations.

Pan-RAS and pan-*KRAS* inhibitors represent a significant advance for treating *KRAS*-mutant gastrointestinal cancers, particularly PDAC, where diverse *KRAS* variants limit the usefulness of allele-specific inhibitors. Daraxonrasib is the most closely watched in this class and has shown meaningful activity in second-line PDAC—outcomes that exceed historical expectations for current therapies. Its toxicity profile, while notable, has been manageable, with dose modifications common but no treatment discontinuations. These findings support the rationale for its ongoing phase 3 evaluation against standard chemotherapy [77,78].

Beyond expanding target coverage across *KRAS* alleles, pan-*KRAS* and pan-RAS inhibitors introduce distinct biological trade-offs compared with allele-selective agents. Unlike mutation-specific inhibitors that exploit structural vulnerabilities unique to mutant *KRAS*, pan-*KRAS* strategies suppress both mutant and wild-type RAS signaling, raising concerns about therapeutic index given the role of wild-type *KRAS* in normal epithelial and hematopoietic function. Early clinical experience with daraxonrasib suggests that partial, context-dependent RAS suppression may be sufficient to impair tumor growth without prohibitive toxicity, as reflected by manageable adverse events despite frequent dose modifications [78]. However, whether this balance can be sustained with prolonged treatment or in combination regimens remains uncertain. Additionally, the tri-complex mechanism of RMC-6236—requiring cyclophilin A engagement—introduces potential interpatient variability related to intracellular protein–protein interactions, which may complicate biomarker development and patient selection [24].

From a resistance perspective, pan-*KRAS* inhibitors may delay—but not eliminate—adaptive escape. By targeting multiple *KRAS* variants simultaneously, these agents could reduce secondary allele switching, a resistance mechanism observed with allele-selective inhibitors [22]. However, broader RAS suppression may intensify selection for non-RAS bypass pathways, including PI3K activation, MYC amplification, MET signaling, or transcriptional lineage switching, all of which are established resistance mechanisms in *KRAS*-driven GI cancers [23,79]. The immunologic consequences of pan-*KRAS* inhibition also remain poorly defined. While *KRAS* blockade can remodel the tumor microenvironment, sustained suppression of wild-type RAS signaling in immune cells could complicate combination strategies with immune checkpoint inhibitors [66,80,81]. Thus, although pan-*KRAS* inhibitors represent a promising advance—particularly for heterogeneous *KRAS*-mutant PDAC—their long-term clinical impact will likely depend on precise dosing, rational combinations, and disease-specific application [76,78,82]. Ongoing clinical development of pan-*KRAS* and RAS(ON) inhibitors in PDAC is detailed in Supplementary Table S3.

Take-Home Message:

Clinical Maturity:

Mid-stage development (Phase I–III). Most advanced pan-RAS agent (daraxonrasib/RMC-6236) undergoing phase III evaluation in PDAC.

Likelihood of Regulatory Approval:

Moderate to high in PDAC if phase III data confirm survival benefit. Broader approval dependent on tolerability and durability.

Expected Role in Treatment:

Most promising near-term strategy for heterogeneous *KRAS*-mutant PDAC. Likely second-line therapy initially, with potential earlier-line incorporation if survival benefit is confirmed. May serve as a backbone for combination regimens.

### 3.5. KRAS Vaccines

Cancer vaccines have been discussed for many years, with several strategies under current clinical development. Amplify-7P is a clinical trial investigating the adjuvant *KRAS*-specific ELI-002 7p vaccine, which comprises amphiphile modified *KRAS* G12 D, C, A, V, R and G13D variant peptides that will ideally stimulate enough T cell response to shrink PDAC and CRC tumors [83]. This trial is made up of PDAC and CRC patients with minimal residual disease (MRD) following standard locoregional treatment. PDAC and CRC patients with elevated circulating tumor DNA (ctDNA) and/or serum tumor biomarkers CA19-9 and CEA were enrolled. Keeping in mind the small sample size (12 patients), ctDNA or CA19-9/CEA reduction/clearance were seen in 40% (2/5) and 71% (5/7) of patients given the 1.4 mg and 4.9 mg doses, respectively, and T cell response was seen in two thirds of patients. A phase II trial is currently underway and recruiting (NCT05726864). In another effort, a *KRAS*-targeted long peptide vaccine is currently being tested with ipilimumab and nivolumab in advanced PDAC and CRC. A phase I trial is underway consisting of a primary phase (vaccine given on days 1, 8, 15, and 22) as well as a boosting phase (vaccine given on weeks 13, 21, 29, and 37) with safety and T cell response as the primary outcomes [84]. A lipid nanoparticle vaccine being developed by Moderna—mRNA-5671 (also known as V941)—is currently being tested in a phase I trial that targets cancers with *KRAS* G12D, G12V, G12C and G13D alterations (NCT03948763). After vaccination, antigen presenting cells (APCs) take up and translate this V941 lipid particle into peptides that are then presented via MHC molecules to stimulate cytotoxic and memory T cells against tumors harboring the *KRAS*-mutated protein [85].

Collectively, these vaccine strategies highlight a promising shift toward immunologic control of *KRAS*-mutant GI cancers, addressing a major historical challenge: *KRAS* mutations produce “self-like” neoantigens that are weakly immunogenic, particularly in immunosuppressive tumors like PDAC [86]. The early results from ELI-002 7P suggest that peptide vaccines can generate measurable immune responses and potentially reduce minimal residual disease, which could translate to delayed recurrence—an area of unmet need in PDAC and CRC [87,88]. However, the extremely small sample size and reliance on surrogate biomarkers (ctDNA, CA19-9, CEA) limit definitive conclusions about clinical benefit. Long-peptide vaccines and mRNA-based vaccines offer advantages in antigen breadth and immunogenicity, but their efficacy will depend heavily on overcoming the dense stroma, poor T-cell infiltration, and immunosuppressive microenvironment characteristic of PDAC and some CRC subtypes [88,89].

Despite early immunologic signals, several biologic and clinical uncertainties temper enthusiasm for *KRAS*-targeted vaccination strategies in gastrointestinal malignancies [86,90]. First, the intrinsic immunologic context of PDAC—characterized by dense desmoplasia, poor T-cell infiltration, suppressive myeloid populations, and cytokine-mediated immune exclusion—raises concern that vaccine-induced T-cell priming may not translate into effective intratumoral cytotoxicity without concurrent microenvironment modulation [37,80,81]. While circulating *KRAS*-specific T-cell responses and reductions in ctDNA or tumor markers are encouraging, these surrogate endpoints do not yet establish durable relapse prevention or overall survival benefit, particularly given the profound stromal and metabolic constraints that limit immune trafficking in PDAC [37,87]. Second, *KRAS*-driven tumors exhibit substantial transcriptional plasticity and adaptive signaling rewiring under therapeutic pressure [72], suggesting that immune-targeted pressure alone may select for antigen-loss variants, MHC downregulation, or non-genetic escape mechanisms. Third, the heterogeneity of *KRAS* alleles across PDAC and CRC—including G12D, G12V, G12R, and others—raises questions regarding breadth of epitope coverage and the feasibility of generating sufficiently robust, allele-spanning immune responses in a genetically diverse patient population [15].

Moreover, the immunologic consequences of combining *KRAS* vaccines with checkpoint inhibitors remain uncertain. Although *KRAS* inhibition can transiently enhance tumor immunogenicity and reduce PD-L1-mediated immune suppression [91], sustained pathway suppression may induce compensatory inflammatory signaling or immune regulatory recruitment that limits durability [80]. The timing, sequencing, and patient selection for vaccine–immunotherapy combinations therefore require careful biomarker-guided optimization rather than empirical layering of agents. Finally, the low tumor mutational burden characteristic of PDAC compared with other solid tumors may inherently limit neoantigen breadth and T-cell clonality, constraining vaccine efficacy relative to more immunogenic malignancies [21]. Collectively, while *KRAS*-directed vaccines represent a rational and innovative strategy—particularly in the minimal residual disease setting—their ultimate clinical impact will likely depend on integration with stromal modulation and pathway inhibition rather than reliance on vaccination alone. Durable benefit will require not only successful immune priming but also sustained intratumoral effector function within a highly suppressive *KRAS*-driven microenvironment. A summary of *KRAS*-directed vaccines, adoptive cellular therapies, and other immunologic strategies in PDAC is provided in Supplementary Table S4.

Take-Home Message:

Clinical Maturity:

Early-phase clinical development with immunologic and minimal residual disease signals; limited survival data.

Likelihood of Regulatory Approval:

Low in the near term; dependent on demonstration of durable clinical endpoints beyond surrogate biomarker responses.

Expected Role in Treatment:

Most plausible in minimal residual disease or adjuvant settings rather than bulky metastatic disease. Likely to require combination with checkpoint inhibitors or *KRAS* pathway inhibition for meaningful clinical impact.

## 4. Challenges of *KRAS* Therapies and Future Directions

### 4.1. KRAS Beyond PDAC and CRC

Discussions around *KRAS*-directed therapeutics in GI malignancies are usually focused on PDAC and CRC, given the high frequencies of *KRAS* alteration in up to 90% and 50% of cases respectively. However, many other GI malignancies harbor *KRAS* alterations, and these diseases also need to be taken into consideration when conducting clinical trials on *KRAS*-altered disease. *KRAS* mutations and amplification are seen in roughly 10–15% of all gastric cancers but that percentage increases to nearly 40% in human-intestinal type gastric cancers [92]. In addition, *KRAS* alterations are seen in close to 50% of appendiceal carcinomas, roughly 20% for both cholangiocarcinoma and gallbladder carcinoma, and between 5 and 10% of esophageal cancers [93,94,95]. Among these GI malignancies, *KRAS* G12D appears to be the most common variant seen [93,96]. There are comparatively fewer pre-clinical datasets describing the influence *KRAS* mutations have on tumor behaviors beyond PDAC and CRC. Mouse model studies of *KRAS*-mutated alleles over the past decade have shown that *KRAS* activating mutations lead to gastric tissue metaplasia and features of carcinoma [95,97]. However, further work is needed to map out carcinogenesis in gastric and other cancers in the setting of activating *KRAS* mutations. Both the updated CodeBreak and KRYSTAL trial analysis for Sotorasib and Adagrasib enrolled only PDAC and CRC *KRAS*-mutated GI cancer patients and current active clinical trials to date have no meaningful sample size of these other *KRAS*-mutated GI malignancies—if any enrollment at all. For example, a phase I study testing Divarasib with G12C mutated cancers enrolled 19 patients who did not have NSCLC, CRC or PDAC out of the 137 total patients [43]. This is likely because of how rare these other malignancies are coupled with their low *KRAS* G12C mutation frequency. As the landscape of *KRAS*-targeted therapy grows, additional pre-clinical research into how *KRAS* mutations shape tumorigenesis and the TME of different GI malignancies, in addition to PDAC and CRC, will support expanded clinical trials for a broader spectrum of GI cancer patients in the future.

### 4.2. Mechanisms of Resistance to KRAS Inhibitors

Resistance to *KRAS*-directed therapy in gastrointestinal malignancies reflects the interaction of *KRAS* allele biology, tumor lineage, and inhibitor class, rather than a single uniform mechanism. In *KRAS* G12C–mutant colorectal cancer, adaptive resistance is predominantly driven by EGFR-mediated MAPK reactivation, restoring ERK signaling despite covalent inhibition of mutant *KRAS* [31,35]. Reactivation of upstream receptor tyrosine kinases and SHP2-dependent signaling sustains pathway output and explains the limited efficacy of G12C monotherapy in CRC and the benefit of the combined *KRAS*–EGFR blockade [31,64,98]. In contrast, resistance in PDAC is less EGFR-dependent and more reflective of intrinsic tumor plasticity, co-mutation context, and heterogeneous bypass signaling, including PI3K engagement and metabolic adaptation [99]. These differences underscore that identical *KRAS* alleles exhibit lineage-specific resistance patterns.

Allele-selective inhibitors are particularly susceptible to on-target genetic escape, including secondary *KRAS* mutations affecting drug binding, amplification of the mutant *KRAS* allele with increased signaling flux, and outgrowth of alternative *KRAS* variants [22,72,74]. Such findings highlight the evolutionary plasticity of *KRAS*-driven tumors under therapeutic pressure. As inhibitors targeting G12D and G12V advance, similar resistance mechanisms—including structural alterations or altered nucleotide cycling—remain plausible [21,64,98]. In contrast, pan-*KRAS* and pan-RAS inhibitors may reduce allele-specific escape but intensify selection for non-RAS bypass pathways, including PI3K activation, MYC amplification, MET signaling, and broader pathway rewiring [24,28,74]. Thus, resistance patterns vary not only by mutation subtype but also by the scope of pathway suppression achieved.

Tumor microenvironment (TME) dynamics further shape resistance in a disease-specific manner. In CRC, resistance often reflects receptor-driven epithelial signaling [30,35]. In PDAC, dense desmoplasia, cancer-associated fibroblasts, metabolic reprogramming, and immune exclusion contribute to impaired drug penetration and sustained survival signaling [65,67,81]. *KRAS* inhibition may transiently enhance tumor immunogenicity; however, sustained blockade can induce compensatory cytokine signaling, immune-suppressive recruitment, and inflammatory feedback that limit durability [67,81]. Additionally, epithelial–mesenchymal transition-like shifts and lineage plasticity programs provide non-genetic routes to escape following MAPK suppression [21,72]. These mechanisms appear particularly relevant in PDAC and support integration of stromal, metabolic, or immune-modulating strategies alongside *KRAS* inhibition.

Resistance trajectories also differ between off-state inhibitors and emerging RAS(ON) or tri-complex agents. Off-state inhibitors rely on nucleotide cycling and may lose efficacy in tumors favoring persistent GTP-bound *KRAS* or altered upstream flux [11,22]. RAS(ON) and pan-*KRAS* inhibitors suppress active signaling irrespective of nucleotide state, potentially overcoming some biochemical resistance mechanisms but introducing broader adaptive pressures, including pathway rewiring and lineage plasticity. Whether these approaches fundamentally alter long-term evolutionary escape or simply redirect it remains uncertain.

**Figure 2:**
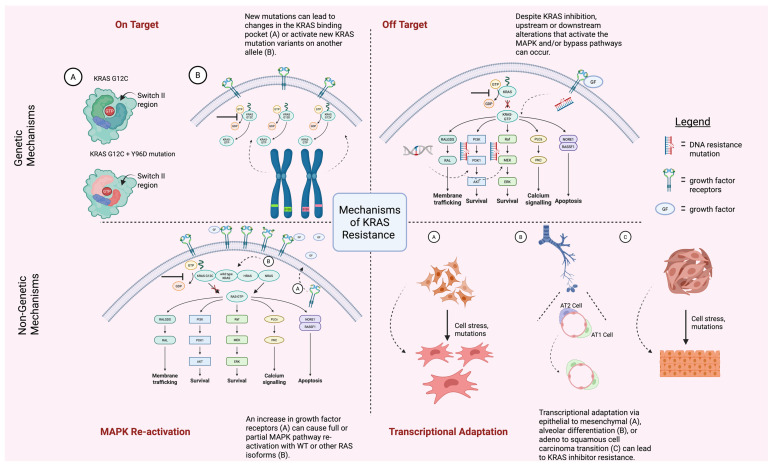
This figure depicts the main mechanisms of *KRAS* resistance. This multi-panel figure is broken down into genetic mechanisms of resistance (top two panels) and non-genetic mechanisms (bottom two panels). Genetic mechanisms of resistance are represented by on target (**top left**) and off target (**top right**) mutations. On target mutations directly involve the *KRAS* gene/protein whereas off target mutations occur in other members of the signaling pathway to help bypass *KRAS* inhibition. On Target (A) depicts a KRAS G12C switch II region with and without a Y96D mutation which causes structural changes to the binding pocket. Once the G12C pathway has been blocked (blunted arrow), On Target (B) depicts activation of new KRAS mutation variants such as G12D (green allele) or G12V (pink allele) and their respective activated pathways (dotted arrows showing the activated signaling pathways corresponding with the new mutated alleles). The Off Target genetic mechanism is depicted by a large signaling pathway beginning with KRAS inhibition upstream (blunted arrow with red X). However, DNA resistance alterations and mutations (red/blue DNA/ladder) can bypass this inhibition and re-activate the pathway (dotted arrows). Non-genetic mechanisms are represented by MAPK re-activation (**bottom left**) and transcriptional adaptation (**bottom right**). MAPK re-activation occurs due to an increase in growth factor receptors on the cell surface (A) in the response to *KRAS* inhibition. Transcriptional adaptation happens when a more *KRAS* inhibitor sensitive tissue phenotype changes to a tissue phenotype that is less *KRAS* sensitive. The bottom right figure uses arrows to depict the change in tissue phenotype seen in A, B and C brought on by cell stress and mutations. Created in BioRender. Frisch, A. (2025) https://BioRender.com/actj21g (URL accessed on 17 November 2025).

Collectively, resistance to *KRAS*-directed therapy in GI malignancies must be interpreted within a defined allele–tumor–drug framework. *KRAS* G12C CRC is primarily constrained by EGFR- and SHP2-mediated feedback; PDAC by stromal biology, metabolic adaptation, immune modulation, and plasticity; and pan-*KRAS* inhibition by pathway-level bypass and transcriptional rewiring. Effective strategies will require biomarker-informed combinations tailored to mutation subtype and tumor context rather than assuming a universal model of *KRAS* inhibitor resistance. Figure 2 centers on the mechanisms that drive resistance to *KRAS*-directed therapies, illustrating how adaptive and acquired escape pathways restore MAPK signaling despite pharmacologic inhibition. The figure also distinguishes on-target genetic escape—such as secondary *KRAS* mutations or mutant allele amplification—from off-target mechanisms. By integrating adaptive feedback, genetic resistance, and lineage-specific bypass signaling, Figure 2 provides a mechanistic framework explaining why single-agent *KRAS* inhibition is frequently transient and why durable responses likely require multi-level pathway suppression.

### 4.3. Drug Delivery Limitations and Failure in PDAC

Despite compelling preclinical evidence identifying *KRAS* as the dominant oncogenic driver in PDAC, clinical attempts to inhibit *KRAS* or its downstream effectors have largely failed, due in part to profound drug delivery barriers intrinsic to PDAC biology [15,16]. PDAC is characterized by a dense desmoplastic stroma composed of cancer-associated fibroblasts, extracellular matrix components, and immune cells, resulting in elevated interstitial fluid pressure, vascular compression, and markedly impaired intratumoral drug perfusion [86,89,100]. Even highly potent small-molecule inhibitors may not achieve uniform or sustained intratumoral concentrations sufficient for durable *KRAS* pathway suppression. These pharmacokinetic limitations help explain the disconnect between strong *KRAS* inhibition observed in genetically engineered mouse models and the consistent failure of indirect *KRAS*-targeting strategies—such as farnesyltransferase, MEK, PI3K, and ERK inhibitors—in late-phase PDAC trials [16]. Heterogeneous drug exposure within the tumor microenvironment may promote the rapid selection of resistant subclones, further limiting therapeutic durability [64]. *KRAS*-driven PDAC also actively remodels its microenvironment to promote immune exclusion, metabolic adaptation, and stromal expansion, reinforcing both physical and functional resistance to therapy [37,66]. Importantly, partial or spatially limited *KRAS* inhibition may be insufficient in PDAC, where residual signaling can sustain tumor growth and survival [21]. Together, these delivery constraints help explain why first-generation *KRAS* inhibitors have produced only modest and transient responses in PDAC despite systemic target engagement. Emerging strategies—including pan-RAS and active-state (“RAS-on”) inhibitors, stromal-modulating combinations, and nanoparticle-based delivery approaches—may help overcome these barriers by improving intratumoral exposure, limiting pathway redundancy, and delaying adaptive resistance. Collectively, the historical failure of *KRAS*-directed therapies in PDAC reflects the uniquely hostile delivery environment of pancreatic tumors, which must be addressed for *KRAS*-targeted strategies to be successful.

### 4.4. Other Signaling Pathway Targets and Combination Strategies

Specific trials and therapeutics have also explored the utility of targeting downstream and upstream MAPK/ERK pathway components. This could have broad application for all tumors influenced by the MAPK/ERK pathway. Upstream epidermal growth factor inhibitors (EGFR), BRAF inhibitors, mitogen-activated protein kinase (MEK) inhibitors, and the pathway regulators have all been targets of early trials as monotherapy and/or as combination therapy with chemotherapy. Early trial data and research in targets such as Src homology-containing protein tyrosine phosphatase 2 (SHP2) and son of sevenless homolog 1 (SOS1) in combination with chemotherapy over the past few years have shown little benefit in overall survival for patients with RAS-mutated tumors [101,102,103]. However, given the importance of these pathways in conjunction with RAS signaling, several of these are being tested in trials with RAS inhibitors. Current clinical trials are ongoing for SHP2 inhibitors for advanced solid tumors, including HBI-2376 (NCT05163028). Clinical trials combining SHP2 and mutant G12C inhibitors are also underway, including adagrasib plus TNO155 (NCT04330664), JAB-21822 plus JAB-3312 (NCT05288205), and BBP-398 plus sotorasib (NCT05480865). BI-1701963 is a small molecule SOS1 inhibitor that is being studied in clinical trials in combination with adagrasib and trametinib (NCT04975256), (NCT04111458). In addition to EGFR and SHP2 signaling, ADAM9 has emerged as an upstream modulator of receptor tyrosine kinase signaling in GI cancers. Experimental studies show that ADAM9 can promote EGFR phosphorylation and downstream ERK activation and that ADAM9 blockade (including with an ADAM9-targeting antibody) suppresses tumor growth in ADAM9-high models [104]. Elevated ADAM9 expression has also been reported in colorectal and pancreatic cancers and correlates with aggressive pathologic features and adverse outcomes, including invasion-related phenotypes in CRC and poor prognostic features in PDAC [105,106]. In colon cancer models, the ADAM9–EGFR axis has been linked to treatment resistance (e.g., fluoropyrimidine resistance), supporting the broader concept that ADAM9-associated EGFR/MAPK signaling may contribute to therapeutic escape [104].

The most recent combination strategy regarding *KRAS* inhibition involves immunotherapy. It has been documented in the literature that *KRAS* mutations influence the TME and creates an immunosuppressive environment [107,108]. Combining *KRAS* inhibitors with immune-checkpoint inhibitors is promising because direct *KRAS* inhibition can partially reverse immunosuppressive tumor microenvironment (TME) features: it reduces PD-L1 expression, normalizes cytokine secretion, and can enhance T-cell infiltration [91]. On vs. Off inhibitor selection may influence the magnitude and timing of immune remodeling; for example, Off inhibitors induce acute suppression of *KRAS*-driven immunosuppressive signaling, creating a window for T-cell activation, while On inhibitors may sustain prolonged pathway inhibition potentially improving synergy with immunotherapy [56]. Researchers have recently taken an interest in studying *KRAS* inhibitors in combination with immune checkpoint inhibitors such as pembrolizumab. The pan-*KRAS* inhibitor RMC-6236 and RMC-6291 are both being tested in combination with pembrolizumab in patients with NSCLC (NCT06162221). A trial is currently active testing sotorasib with AMG404 for advanced solid tumors with a G12C mutation (NCT04185883). These current trials represent the future of treatment beyond *KRAS* monotherapy, with the goal of creating a suite of combination therapies that will mitigate resistance mechanisms and give patients more options upon first-line therapy resistance.

## 5. Conclusions

*KRAS*-directed therapy in gastrointestinal malignancies has transitioned from theoretical possibility to clinical reality, yet its impact varies substantially by allele, tumor lineage, and therapeutic strategy. In the near term, the most clinically meaningful advances are likely to arise from rational combination approaches rather than monotherapy. In colorectal cancer, vertical pathway inhibition—particularly dual *KRAS* G12C and EGFR blockade—has already established proof of principle that resistance can be delayed through feedback suppression. In pancreatic ductal adenocarcinoma, where *KRAS* mutations are nearly ubiquitous but biologically heterogeneous, pan-*KRAS* and RAS(ON) inhibitors currently represent the most promising strategy to address allelic diversity and reduce on-target escape. However, durable benefit will almost certainly require integration with stromal modulation, immune-directed therapy, or downstream pathway inhibition to overcome adaptive signaling and microenvironmental resistance. Thus, biomarker-guided combinations tailored to mutation subtype and tumor context represent the most realistic path to incremental survival gains over the next several years.

Longer-term experimental strategies—including allele-specific G12D and G12V inhibitors, *KRAS*-directed vaccines, T-cell receptor therapies, and targeted protein degraders—remain scientifically compelling but should be viewed as developmental platforms rather than near-term standards of care. Their ultimate success will depend on demonstrating durability, defining resistance trajectories, and identifying optimal combination partners. Future progress will require moving beyond a mutation-centric framework toward an integrated allele–tumor–drug model that accounts for lineage-specific biology, co-mutation context, stromal architecture, and immune landscape. Aligning therapeutic intensity with *KRAS* biology—rather than applying uniform strategies across PDAC, CRC, and rarer GI malignancies—offers the clearest path forward. The field has entered a transformative phase. Sustained clinical impact will depend not only on improving *KRAS* inhibition itself, but on strategically embedding these agents within rational, disease-specific combination paradigms.

## Data Availability

No new data were created or analyzed in this study.

## Supplementary Materials

The following supporting information can be downloaded at: https://www.mdpi.com/article/10.3390/curroncol33030148/s1, Table S1: Clinical development landscape of *KRAS*-targeted therapies in non-pancreatic gastrointestinal malignancies, primarily colorectal cancer (CRC). Table S2: Ongoing clinical development of allele-specific *KRAS* inhibitors and cellular therapies in pancreatic ductal adenocarcinoma (PDAC). Table S3: Pan-*KRAS* and RAS(ON) inhibitor platforms under clinical investigation in pancreatic ductal adenocarcinoma. Table S4: *KRAS*-directed immunotherapeutic strategies in pancreatic ductal adenocarcinoma, including vaccines, adoptive cellular therapies, and novel biologic approaches.

## Abbreviations

APCs	Antigen-presenting cells
AT1/AT2	Alveolar type 1/type 2 cells
BEACON	BEACON CRC trial (encorafenib + cetuximab)
BRAF	v-Raf murine sarcoma viral oncogene homolog B1
CAF(s)	Cancer-associated fibroblast(s)
CA19-9	Carbohydrate antigen 19-9
CEA	Carcinoembryonic antigen
CRC	Colorectal cancer
ctDNA	Circulating tumor DNA
DCR	Disease control rate
EGFR	Epidermal growth factor receptor
EMT	Epithelial–mesenchymal transition
ERK	Extracellular signal-regulated kinase
GTPase	Guanosine triphosphatase
GTP	Guanosine triphosphate
GDP	Guanosine diphosphate
HRAS	Harvey rat sarcoma viral oncogene homolog
*KRAS*	Kirsten rat sarcoma viral oncogene homolog
MAPK	Mitogen-activated protein kinase
mCRC	Metastatic colorectal cancer
MEK	Mitogen-activated protein kinase kinase (MAP2K)
mOS	Median overall survival
mPFS	Median progression-free survival
MHC	Major histocompatibility complex
MRD	Minimal residual disease
MYC	MYC proto-oncogene
NSCLC	Non-small cell lung cancer
ORR	Objective response rate
PDAC	Pancreatic ductal adenocarcinoma
PFS	Progression-free survival
PI3K	Phosphatidylinositol-3-kinase
PROTAC	Proteolysis-targeting chimera
RAF	Rapidly accelerated fibrosarcoma kinase family
RAS	Rat sarcoma (small GTPase family)
SHP2	Src homology 2 domain-containing phosphatase 2 (PTPN11)
SOS1	Son of sevenless homolog 1
TCR	T-cell receptor
TME	Tumor microenvironment
TRAE(s)	Treatment-related adverse event(s)
