# Supplementary material for: Unlocking KRAS: Navigating Its Molecular Biology and Treatment Landscape Among Gastrointestinal Malignancies
Source: Curr Oncol. 2026 Mar 3;33(3):148. doi: 10.3390/curroncol33030148 (PMC13024909; doi:10.3390/curroncol33030148)
Supplement: Supplementary file 1 [file curroncol-33-00148-s001.zip › curroncol-4120000-supplementary.pdf]

Table S1. Clinical development landscape of KRAS-targeted therapies in non-pancreatic gastrointestinal malignancies, primarily colorectal cancer (CRC).

Class	Agent / Study	NCT ID	Phase	Notes
**G12C inhibitors (monotherapy)**	BPI-421286	NCT05315180	Phase 1	Early agent
	GFH925	NCT05005234	Phase 1/2	Advanced tumors
	Sotorasib (CodeBreak 101)	NCT04185883	Phase 1	Foundational
	GDC-6036	NCT04449874	Phase 1	Combination-ready
	JDQ443	NCT04699188	Phase 1/2	Platform trial
	ZG19018 (Adagrasib)	NCT06237400	Phase 1/2	Variant
**G12C + EGFR combinations**	IBI351 + cetuximab	NCT05497336	Phase 1	CRC-specific
	JAB-21822 + cetuximab	NCT05194995	Phase 1/2	Combination
	Adagrasib + cetuximab ± irinotecan	NCT05722327	Phase 1	Triplet strategy
	Adagrasib + cetuximab + cemiplimab	NCT06412198	Phase 1/2	IO combination
**Phase 3 CRC trials**	Sotorasib + panitumumab	NCT05198934	Phase 3	Practice-changing
	Adagrasib + cetuximab (KRYSTAL-10)	NCT04793958	Phase 3	Confirmatory
	Sotorasib + FOLFIRI ± bevacizumab	NCT06252649	Phase 3	Frontline setting
**G12D inhibitors**	TSN1611	NCT06385925	Phase 1/2	Emerging
	ASP4396	NCT06364696	Phase 1	Early phase
**Pan-KRAS inhibitors**	YL-17231	NCT06078800	—	Broad KRAS
	BBO-10203 (BREAKER-101)	NCT06625775	Phase 1	Next-gen
**Other strategies**	MRTX0902 (MAPK pathway)	NCT05578092	Phase 1/2	Pathway targeting
	Dual ON/OFF inhibitor (BBO-8520)	NCT06343402	Phase 1	Novel mechanism
	TCR-T + vaccine	NCT06253520	Phase 1	Cellular + vaccine

Table S1. Clinical development landscape of KRAS-targeted therapies in non-pancreatic gastrointestinal malignancies, primarily colorectal cancer (CRC).
This table outlines mutation-selective KRAS inhibitors, combination strategies with EGFR-directed therapy, pan-KRAS inhibitors, and other pathway-targeting approaches in CRC and related GI tumors. Included are monotherapy G12C inhibitors, KRAS G12C plus EGFR antibody combinations, frontline and refractory Phase 3 confirmatory trials, emerging G12D inhibitors, and next-generation pan-KRAS/RAS(ON) inhibitors. Cellular and vaccine-based combinatorial strategies are also summarized. Trial phase and NCT identifiers are provided to contextualize clinical maturity.

Table S2. Ongoing clinical development of allele-specific KRAS inhibitors and cellular therapies in pancreatic ductal adenocarcinoma (PDAC).

Target	Agent / Study	NCT ID	Phase	Notes
**G12C inhibitors**	HBI-2438	NCT05485974	Phase 1	Monotherapy solid tumors
	JAB-21822	NCT06008288	Phase 2	PDAC-specific
	JAB-21822 + JAB-3312	NCT05288205	Phase 1/2	Combination strategy
	RMC-6291	NCT05462717	Phase 1	RAS(ON) inhibitor
	RMC-6291 + RMC-6236	NCT06128551	Phase 1	Combination with pan-RAS
	LY3537982	NCT04956640	Phase 1/2	Advanced solid tumors
	Adagrasib (MRTX849)	NCT05634525	Phase 1	PDAC-specific cohort
	FMC-376	NCT06244771	Phase 1/2	Advanced solid tumors
**G12D inhibitors**	MRTX1133	NCT05737706	Phase 1/2	Key PDAC-relevant agent
	QLC1101	NCT06403735	Phase 1	Early phase
	RMC-9805	NCT06040541	Phase 1	Emerging agent
	AZD0022	NCT06599502	Phase 1/2	Mono + combination
	LY3962673	NCT06586515	Phase 1	Early phase
**Other allele-specific (cellular)**	G12V TCR-T (FH-A11KRASG12V)	NCT06043713	Phase 1	TCR-engineered
	G12V TCR lymphocytes	NCT03190941	Phase 1/2	HLA-restricted
	G12D TCR lymphocytes	NCT03745326	Phase 1/2	HLA-restricted
	NT-112 (G12D TCR)	NCT06218914	Phase 1	Highly specific

Table S2. Ongoing clinical development of allele-specific KRAS inhibitors and cellular therapies in pancreatic ductal adenocarcinoma (PDAC).

This table summarizes small-molecule KRAS G12C and G12D inhibitors, as well as adoptive T-cell receptor (TCR)-engineered cellular therapies targeting specific KRAS mutations in PDAC. Agents are organized by mutation specificity, trial identifier (NCT number), development phase, and key study features. G12C-directed therapies include both covalent inhibitors and RAS(ON) platform agents, while G12D programs highlight emerging mutation-selective compounds currently in early-phase evaluation. Cellular strategies include HLA-restricted TCR-T approaches targeting KRAS G12V and G12D mutations. Phases reflect trial status at time of manuscript preparation.

Table S3. Pan-KRAS and RAS(ON) inhibitor platforms under clinical investigation in pancreatic ductal adenocarcinoma.

Class	Agent / Study	NCT ID	Phase	Notes
**Pan-KRAS inhibitors**	BGB-53038	NCT06585488	Phase 1	First-in-human
	PF-07934040	NCT06447662	Phase 1	Mono + combination
	LY4066434	NCT06607185	Phase 1	Broad KRAS targeting
**RAS(ON) inhibitors**	RMC-6236	NCT05379985	Phase 1	Foundational agent
	RMC-6236 (Phase 3 PDAC)	NCT06625320	Phase 3	Most advanced PDAC trial
	RAS(ON) inhibitor platform	NCT06445062	Phase 1/2	GI malignancies

Table S3. Pan-KRAS and RAS(ON) inhibitor platforms under clinical investigation in pancreatic ductal adenocarcinoma.

This table details first-in-human and advanced-phase trials evaluating mutation-agnostic KRAS inhibitors and RAS(ON)-state selective compounds in PDAC. Agents include broad-spectrum pan-KRAS inhibitors targeting multiple KRAS variants as well as RAS(ON) inhibitors designed to bind active GTP-bound KRAS. Ongoing Phase 1–3 trials are listed with NCT identifiers and key design features. The inclusion of a Phase 3 PDAC trial highlights the maturation of RAS(ON) inhibition strategies in this disease**.**

Table S4. KRAS-directed immunotherapeutic strategies in pancreatic ductal adenocarcinoma, including vaccines, adoptive cellular therapies, and novel biologic approaches.

Modality	Agent / Study	NCT ID	Phase	Notes
**Vaccines**	ELI-002 (2P)	NCT04853017	Phase 1	Amph-peptide
	ELI-002 (7P)	NCT05726864	Phase 1/2	Multi-allele targeting
	KRAS peptide vaccine + nivolumab/ipilimumab	NCT04117087	Phase 1	Combination IO
	KRAS peptide vaccine (high-risk PDAC)	NCT05013216	Phase 1	Prevention setting
	ATP150/152 + ezabenlimab (KISIMA-02)	NCT05846516	Phase 1	Vaccine + IO
	KRAS mRNA vaccine (ABO2102)	NCT06577532	Early Phase 1	Neoantigen-based
	KRAS vaccine + balstilimab/botensilimab	NCT06411691	Phase 1	Dual checkpoint
**Cellular therapies**	G12V TCR-T (mutant KRAS)	NCT04146298	Phase 1/2	PDAC-specific
	IX001 TCR-T	NCT06487377	Phase 1	Multi-cancer
	Engineered TCR T cells (G12V)	NCT06105021	Phase 1/2	Advanced tumors
**Other approaches**	KRAS G12D siRNA exosomes	NCT03608631	Phase 1	Novel delivery
	Anti-CD38 + KRAS vaccine + PD-1	NCT06015724	Phase 2	Triplet immunotherapy

Table S4. KRAS-directed immunotherapeutic strategies in pancreatic ductal adenocarcinoma, including vaccines, adoptive cellular therapies, and novel biologic approaches.
This table summarizes peptide vaccines, mRNA-based neoantigen vaccines, combination immune checkpoint strategies, TCR-engineered T-cell therapies, and KRAS-targeted siRNA delivery platforms in PDAC. Programs are categorized by modality, with trial identifiers and development phase included. Strategies range from early-phase safety studies to combinatorial immunotherapy approaches integrating checkpoint blockade or antibody-based therapies, reflecting efforts to enhance KRAS-specific immune targeting in pancreatic cancer.
